# Impact of temperature trend-defined seasonality on psoriasis treatment outcomes: a multicenter longitudinal study

**DOI:** 10.3389/fimmu.2025.1641225

**Published:** 2025-09-17

**Authors:** Xinyi Song, Qin Yang, Yuye Wang, Ning Yu

**Affiliations:** ^1^ Department of Dermatology, Shanghai Skin Disease Hospital, Institute of Psoriasis, Tongji University School of Medicine, Shanghai, China; ^2^ Department of Dermatology, Huashan Hospital, Fudan University, Shanghai, China

**Keywords:** psoriasis, epidemiology, temperature, treatment, outcome

## Abstract

**Background:**

Psoriasis severity and symptoms are widely known to vary seasonally. However, evidence on the impact of seasonality on treatment outcomes is limited, with vague season definitions. It also remains unclear whether seasons represent static meteorological levels or dynamic trends.

**Objective:**

To assess the impact of a novel temperature trend-defined seasonality on psoriasis treatment responses at 2 and 3 months.

**Methods:**

Data were derived from the Shanghai Psoriasis Effectiveness Evaluation CoHort (SPEECH), a prospective, multicenter registry assessing the effectiveness of biologics (adalimumab, ustekinumab, secukinumab and ixekizumab), conventional systemic therapies (acitretin and methotrexate), and phototherapy. Patients were categorized into warming (consistent temperature increase), transition (non-unidirectional changes), and cooling (consistent temperature decrease) groups based on ambient temperature trends during the treatment period. Effectiveness was defined as achieving Psoriasis Area and Severity Index (PASI) 75 (≥ 75% improvement in PASI), PASI 90 (≥ 90% improvement in PASI), Physician’s Global Assessment (PGA) of 0/1, and Dermatology Quality of Life Index minimal important difference (DLQI MID) (≥ 4 points improvement) at 2 and 3 months. Covariate balancing propensity score (CBPS) weighting was applied to balance baseline covariates, and odds ratios (ORs) with 95% confidence intervals (CIs) were estimated. Interaction analyses evaluated potential factors that may stratify treatment response.

**Results:**

In the 3-month analysis of 1411 patients, the cooling group showed significantly lower odds of achieving PASI 75 (adjusted OR 0.70, 95% CI 0.61–0.80, *P* <.001), PASI 90 (adjusted OR 0.68, 95% CI 0.59–0.79, *P* <.001), PGA 0/1 (adjusted OR 0.65, 95% CI 0.57–0.75, *P* <.001), and DLQI MID (adjusted OR 0.86, 95% CI 0.75–0.99, *P* = .032) compared to the warming group. The transition group showed intermediate outcomes. Body mass index (BMI) significantly modified treatment effectiveness, with higher BMI associated with poorer responses, whereas treatment type did not alter the seasonal effect. Findings were largely consistent at 2 months.

**Conclusions:**

Cooling trends are associated with reduced treatment efficacy independently of static temperature, humidity, and ultraviolet levels. This BMI-modified effect underscores the importance of personalized management strategies addressing both environmental and patient-specific factors.

## Highlights

- Psoriasis severity is widely recognized, with evidence showing it exhibits seasonal variation.
- Evidence comparing the effectiveness of systemic therapies in psoriasis patients across different seasons is lacking.
- It remains unclear whether the influence of seasons on psoriasis is driven by static meteorological levels or dynamic trends.
- This study introduced a novel classification of seasons based on temperature trends, categorizing the treatment period into warming, transition, and cooling groups.
- Patients in the cooling group exhibited reduced treatment effectiveness, independent of temperature, humidity, and ultraviolet levels during treatment.
- Patients with higher body mass index (BMI) showed a poorer response to the cooling season relative to the warming season, compared to patients with lower BMI.

## Introduction

Psoriasis is a chronic, recurrent inflammatory skin disease affecting 2% to 4% of the global population, with prevalence varying significantly across regions and countries (1). While the pathogenesis of psoriasis remains unclear, environmental factors play a substantial role in triggering and exacerbating the disease (2, 3). These factors include infections (4), lifestyle (5), medications (6), and seasonality (7). Investigating these environmental risk factors and their influence on disease severity, progression, and treatment outcomes is crucial for guiding patient-centered, individualized care and advancing understanding of psoriasis pathogenesis.

Seasonality is widely believed to influence psoriasis. In the United States, dermatology visits for psoriasis increase by 50% in winter compared to summer (8). Similarly, a New England study observed symptom improvement in summer and worsening in winter (9). Globally, internet searches for psoriasis peak in late winter or early spring and decline by late summer (10). However, this seasonal pattern is not consistently supported across studies (11, 12). Most existing research is descriptive, and longitudinal data on the impact of seasonality on systemic therapy effectiveness for psoriasis remain scarce.

The lack of standardized season definitions complicates the interpretation of seasonal effects on psoriasis. Calendar-based methods tied to fixed dates often fail to reflect actual climatic changes or regional variability. To accurately assess the impact of seasonality on treatment outcomes in psoriasis patients, seasons must be precisely defined. Additionally, it remains unclear whether seasons represent static meteorological factors, such as average temperature, ultraviolet (UV) radiation and humidity, or dynamic trends over time. While static measures offer snapshots of conditions, they may overlook the impact of changes influencing disease progression, whereas dynamic trends better capture the environmental stressors affecting patients.

The Shanghai Psoriasis Effectiveness Evaluation CoHort (SPEECH) is a prospective, multicentre, observational registry designed to evaluate the clinical outcomes and safety of phototherapy, conventional systemic therapies, and biologics in patients with psoriasis (13). Using data from SPEECH, this study introduces a novel method of defining seasonality based on annual temperature variation trends. By categorizing treatment windows into warming, transition, and cooling groups, we aim to evaluate the influence of temperature trend-defined seasonality on treatment outcomes in psoriasis patients.

## Methods

### Data source

The design and follow-up protocol of the Shanghai Psoriasis Effectiveness Evaluation CoHort (SPEECH) have been previously described (13). SPEECH is a prospective, multicentre registry established in 2020 to evaluate the comparative effectiveness of systemic therapies and phototherapy in patients with chronic plaque psoriasis. Ethical approval was obtained from the Institutional Review Boards of all participating sites, and all participants provided written informed consent.

### Study population and design

Patients enrolled in the SPEECH registry between November 2020 and June 2023 were included. Eligible participants were ≥ 18 years old, had chronic plaque psoriasis with PASI ≥ 5, and were receiving monotherapy with acitretin, methotrexate, phototherapy, adalimumab, ustekinumab, guselkumab, secukinumab, or ixekizumab. Treatment allocation was not randomized; therapy selection reflected real−world clinical practice, based on disease severity, comorbidities, guideline recommendations, and patient preference.

To ensure treatment−naïve conditions, patients with conventional systemic therapy or phototherapy within the previous 4 weeks or biologic therapy within the previous 12 weeks were excluded. Only patients with a minimum follow−up of 2 or 3 months were included in the analysis, ensuring ≥ 1 month of active treatment exposure.

Patients were enrolled at treatment initiation and followed prospectively according to the registry protocol across seven dermatology centers in Shanghai, China. Standard follow−up visits occurred at 4, 8, 12, 20, 28, 36, 44, and 52 weeks after enrollment. PASI, PGA, and DLQI were assessed at each visit, and PASI scoring was conducted by a dedicated assessment group composed of trained psoriasis specialists at each site, ensuring consistency and reliability across centers.

Each patient was enrolled once per treatment episode. Patients who switched systemic therapy could be re−enrolled as a new treatment episode, with the registry using unique enrollment IDs linked to national ID numbers to prevent duplicate enrollment. For this analysis, switching to a new therapy during follow−up did not result in a separate analytic entry, ensuring that no patient was analyzed multiple times.

The primary endpoint of this cohort study was the proportion of patients achieving PASI 75 (≥ 75% improvement from baseline) at 12 weeks (3 months). Secondary endpoints included PASI 90, PGA 0/1 (clear or almost clear skin), and DLQI minimal important difference (MID, ≥ 4−point reduction), evaluated at 12 and 52 weeks. Absolute PASI < 3 was analyzed as a post hoc exploratory endpoint, as absolute thresholds provide complementary insight into disease control in heterogeneous real−world populations.

If treatment goals were not achieved, clinicians could adjust therapy according to real−world clinical judgment, including dose optimization or interval shortening, switching to a biologic with a different mechanism of action, or initiating combination therapy (e.g., adding phototherapy or a conventional systemic agent). These strategies ensured that treatment was personalized and responsive to individual patient outcomes.

### Temperature data collection

**Figure 1:**
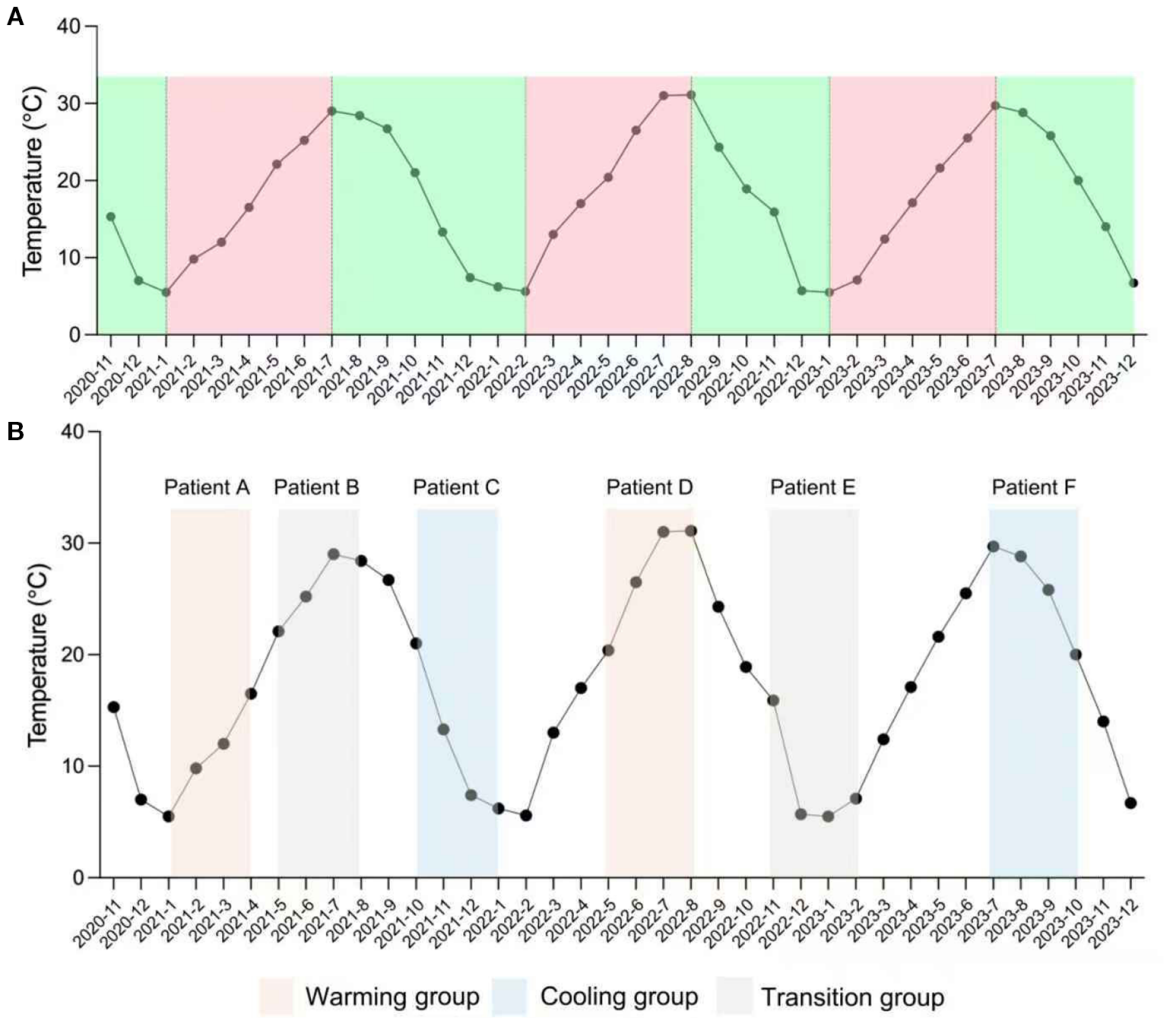
Definition of warming, cooling, and transition groups. **(A)** Monthly mean temperature during the actual study period (November 2020 – December 2023). **(B)** Example of patient assignment to exposure groups using a 12−week analysis window. Only patients with both a defined baseline and 12−week follow−up date were included. Each patient’s treatment period was mapped to the temperature curve to determine the exposure group: Patients A and D fall into the warming group, Patients C and F into the cooling group, and Patients B and E into the transition group.

The periodic temperature variations during the SPEECH study (2020–2023) were sourced from the National Meteorological Information Center (https://data.cma.cn/), providing monthly ambient temperature records for Shanghai (31°12’N, 121°30’E). These data were utilized to analyze temperature trends throughout the treatment period. **Figure 1A** presents a schematic diagram of monthly temperature trends, illustrating how the year can be broadly divided into a temperature-increasing and a temperature-decreasing season based on annual variation patterns.

### Exposure

Patients were categorized into warming, transition, and cooling groups based on temperature trends during the 2- and 3-month treatment windows. The warming group included patients whose treatment windows showed strictly monotonic increases in monthly mean temperature across consecutive months. The cooling group included those with strictly monotonic decreases over the same period. The transition group comprised patients whose windows exhibited any non-monotonic pattern, such as an increase followed by a decrease or vice versa. Because treatment start and end dates could be directly matched to the corresponding meteorological data, each patient’s exposure window was manually assigned to the appropriate category to ensure accurate classification (**Figure 1B**).

### Outcomes

Clinical outcomes were assessed using PASI, the Physician’s Global Assessment (PGA, scored 0 to 4), and the Dermatology Life Quality Index (DLQI), a 10-item patient-reported questionnaire with a total score range of 0 to 30. Key measures included PASI 75 (≥ 75% improvement in PASI), PASI 90 (≥ 90% improvement in PASI), a PGA score of 0 or 1 (indicating clear or almost clear skin), and a DLQI minimal important difference (MID), defined as a reduction of ≥ 4 points. These outcomes were evaluated at 2- and 3-month visits to determine treatment effectiveness and quality of life improvements. In addition, absolute PASI < 3 was evaluated as an exploratory endpoint, as absolute thresholds are particularly informative in real−world registry studies with heterogeneous baseline severity and variable treatment histories.

### Covariates

We considered a broad range of potential confounders, including age at treatment initiation, sex, body mass index (BMI), disease duration (calculated from treatment initiation), treatment history, such as prior use of biologics, non-biologic systemic therapies, and phototherapy, smoking status, family history of psoriasis, psoriatic arthritis, comorbidities including cardiovascular disease, diabetes, non-alcoholic fatty liver disease (NAFLD), hypertension, hyperlipidemia, and hyperuricemia, baseline PASI, PGA, DLQI scores, as well as mean temperature, mean UV index, and mean humidity during treatment.

### Statistical analysis

Baseline patient and meteorological characteristics were compared across exposure groups using absolute standardized differences, with a threshold of > 0.1 indicating imbalance. Covariate balancing propensity score (CBPS) weighting, implemented via the “CBPS” R package (14, 15), was applied to address baseline imbalances. The CBPS model included covariates such as age, sex, BMI, disease duration, treatment history (prior use of biologics, non-biologic systemic therapies, phototherapy), smoking status, family history of psoriasis, psoriatic arthritis, comorbidities (e.g., cardiovascular disease, diabetes, NAFLD, hypertension, hyperlipidemia, hyperuricemia), baseline PASI, PGA, and DLQI scores, as well as mean temperature, mean UV index, and mean humidity during treatment. Mean temperature and mean UV index were not included simultaneously as independent variables in the CBPS model due to multicollinearity. After weighting, standardized differences were reassessed to confirm covariate balance.

Generalized linear models with a logit link were used, incorporating CBPS weighting, to estimate odds ratios (ORs) with 95% confidence intervals (CIs). To further interpret effect sizes, risk differences (RDs) and numbers needed to treat (NNTs) were calculated for PASI 75, PASI 90, PGA 0/1, and DLQI MID outcomes at 2 and 3 months (16). RDs and their 95% CIs were derived using generalized linear models with an identity link function, with NNTs calculated as the reciprocal of the RDs.

### Sensitivity analyses

A series of sensitivity analyses were performed for PASI 75, PASI 90, PGA 0/1, and DLQI MID outcomes at 2 and 3 months to ensure robustness: (1) Replacing mean temperature with mean UV index in the CBPS model; (2) Truncating CBPS weights at the 5^th^ and 95^th^ percentiles; (3) Excluding patients undergoing phototherapy; (4) Excluding patients exposed to mean temperatures ≤ 10^th^ percentile; and (5) Excluding patients with baseline PASI scores in the ≥ 90^th^ percentile.

### E-value

To evaluate the robustness of findings against potential unmeasured confounders, we calculated the E-value, representing the minimum strength of association, on the risk ratio scale, that an unmeasured confounder would require with both the exposure and outcome to fully explain the observed results after adjusting for covariates (17). For context, the E-value was compared to the strength of associations for select observed covariates. Calculations were performed using the ‘EValue’ package (Version 4.1.3) in R.

### Interaction analysis

Interactions between temperature trend (the cooling group vs. the warming group) and other covariates for PASI 75 at 2 and 3 months were analyzed to assess potential effect modification. Effect modification was assessed on the multiplicative scale using the ratio of ORs and on the additive scale using differences in RDs. All analyses were conducted using multivariable regression analysis with explicit adjustment for all covariates to ensure robust and unbiased estimates.

### Multiple imputation

To address missing data, multiple imputation by chained equations (MICE) was applied to generate 20 imputed datasets, including missing outcomes (PGA 0/1 and DLQI MID) and baseline covariates (18). Each imputed dataset was independently analyzed using the CBPS approach. The results from all 20 imputed datasets were then pooled using Rubin’s rules to produce a single set of adjusted OR estimates, standard errors, and 95% CIs, ensuring robustness and reliability despite incomplete data. The multiple imputation analysis was carried out using the 'mice' package (Version 3.16.0) and 'mitools' (Version 2.4) in R.

All statistical analyses were conducted with R (version 4.4.1, R Foundation for Statistical Computing, Vienna, Austria). A *P*-value of less than 0.05 was regarded as statistically significant. To control the family-wise type I error rate at the 5% level, the Hommel method was applied to adjust the *P*-values for the outcomes assessed at 3 months (19).

## Results

### Baseline characteristics

**Figure 2:**
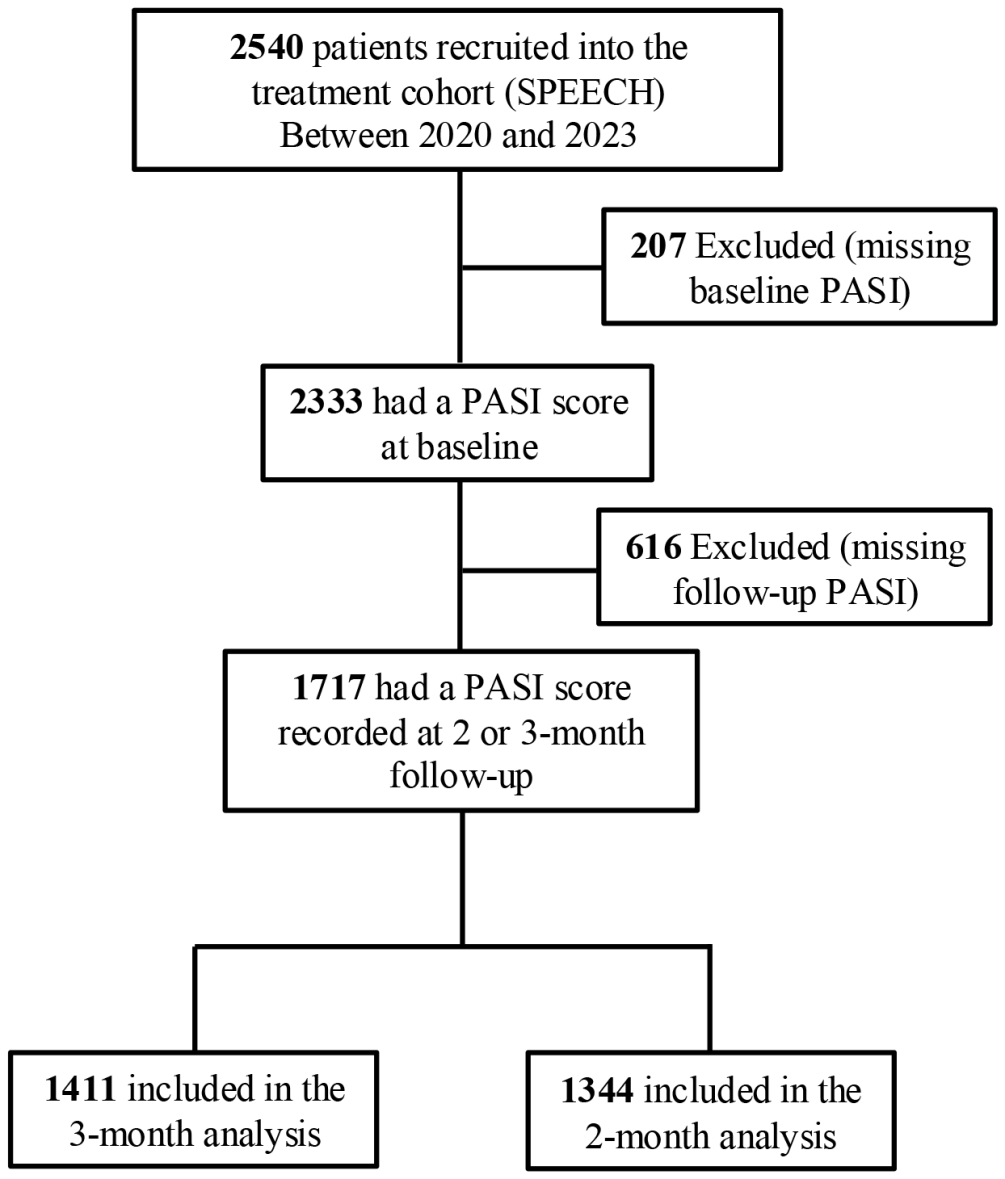
Identification of eligible patients and development of cohorts in the study. SPEECH, Shanghai Psoriasis Effectiveness Evaluation CoHort; PASI, Psoriasis Area and Severity Index.

In the 3-month analysis, a total of 1411 psoriasis patients were included (**Figure 2**), distributed into warming (n = 313), transition (n = 548), and cooling (n = 550) groups based on temperature trends. Before CBPS adjustment, notable imbalances were observed in meteorological metrics, including mean temperature, UV index, and humidity during treatment. Other variables, such as exacerbation seasons and biologic treatment types, showed moderate imbalances. After CBPS weighting, all covariates achieved precise balance, with absolute standardized differences reduced to less than 0.001, ensuring comparability across groups (**Table 1**). Baseline characteristics of patients included in the 2-month analysis are provided in **Supplementary Table S1** (see **Supplementary Material**). Missing data for baseline covariates were below 10%, and there was no missing data for PASI 75 and PASI 90 outcomes (**Supplementary Table S2**; see **Supplementary Material**).

**Table 1:** Patient demographic and clinical characteristics at baseline.

Characteristics	Unweighted population				Weighted population^a,b^		
	Warming	Transition	Cooling	Absolute standardized difference^c^	Warming	Transition	Cooling
Total, n	313	548	550				
Age, years	49.8 (15.4)	48.6 (14.8)	50.1 (16.0)	0.098	49.8 (15.7)	49.8 (14.7)	49.8 (16.6)
Male sex, n (%)	236 (75.4)	395 (72.1)	425 (77.3)	0.052	75.2	75.2	75.2
BMI, kg/m^2^	25.0 (3.4)	24.8 (3.8)	24.6 (3.8)	0.128	24.7 (3.2)	24.7 (3.8)	24.7 (4.0)
College education, n (%)	142 (45.4)	291 (53.1)	257 (46.7)	0.072	49.6	49.6	49.6
Smoker, ex and current, n (%)	152 (48.6)	239 (43.6)	286 (52.0)	0.084	48.3	48.3	48.3
Disease duration, years	15.0 (12.6)	14.0 (11.2)	14.6 (12.3)	0.079	14.6 (12.6)	14.6 (11.9)	14.6 (12.2)
Family history, n (%)	81 (25.9)	128 (23.4)	128 (23.3)	0.026	23.4	23.4	23.4
Psoriatic arthritis, n (%)	25 (8.0)	59 (10.8)	62 (11.3)	0.033	10.5	10.5	10.5
Comorbidities, n (%)							
Cardiovascular disease	17 (5.4)	23 (4.2)	29 (5.3)	0.012	4.8	4.8	4.8
Diabetes	40 (12.8)	59 (10.8)	70 (12.7)	0.020	12.6	12.6	12.6
Hypertension	92 (29.4)	119 (21.7)	146 (26.5)	0.077	25.7	25.7	25.7
NAFLD	42 (13.4)	57 (10.4)	52 (9.5)	0.040	10.5	10.5	10.5
Hyperlipidemia	43 (13.7)	47 (8.6)	65 (11.8)	0.052	10.8	10.8	10.8
Hyperuricemia	24 (7.7)	32 (5.8)	53 (9.6)	0.036	7.9	7.9	7.9
Prior treatment, n (%)							
Biologics	36 (11.5)	35 (6.4)	52 (9.5)	0.049	9.4	9.4	9.4
Systemic nonbiologics	109 (34.8)	195 (35.6)	208 (37.8)	0.030	37.5	37.5	37.5
Phototherapy	138 (44.1)	234 (42.7)	239 (43.5)	0.012	43.1	43.1	43.1
Exacerbation season, n (%)							
Spring	40 (12.8)	61 (11.1)	58 (10.5)	0.026	11.4	11.4	11.4
Summer	13 (4.2)	32 (5.8)	48 (8.7)	0.046	6.5	6.5	6.5
Autumn	35 (11.2)	98 (17.9)	117 (21.3)	0.095	17.5	17.5	17.5
Winter	204 (65.2)	330 (60.2)	293 (53.3)	0.127	58.7	58.7	58.7
Baseline PASI	14.4 (8.8)	14.0 (7.7)	13.9 (8.3)	0.053	14.0 (9.0)	14.0 (7.7)	14.0 (8.2)
Baseline PGA, n (%)							
1 = Minimal	8 (2.6)	22 (4.0)	23 (4.2)	0.016	4.0	4.0	4.0
2 = Mild	90 (28.8)	217 (39.6)	234 (42.5)	0.138	37.8	37.8	37.8
3 = Moderate	147 (47.0)	240 (43.8)	232 (42.2)	0.048	43.3	43.3	43.3
4 = Severe	68 (21.7)	69 (12.6)	61 (11.1)	0.106	14.9	14.9	14.9
Baseline DLQI	9.5 (6.0)	9.8 (6.7)	9.4 (6.7)	0.060	9.4 (6.2)	9.4 (6.6)	9.4 (6.6)
Treatment, n (%)							
Acitretin	35 (11.2)	48 (8.8)	82 (14.9)	0.062	11.8	11.8	11.8
Methotrexate	75 (24.0)	111 (20.3)	134 (24.4)	0.041	23.2	23.2	23.2
Phototherapy	70 (22.4)	108 (19.7)	136 (24.7)	0.050	21.2	21.2	21.2
Adalimumab	6 (1.9)	18 (3.3)	35 (6.4)	0.045	4.5	4.5	4.5
Ustekinumab	20 (6.4)	57 (10.4)	30 (5.5)	0.050	7.9	7.9	7.9
Guselkumab	39 (12.5)	14 (2.6)	8 (1.5)	0.110	4.3	4.3	4.3
Secukinumab	41 (13.1)	131 (23.9)	76 (13.8)	0.108	17.6	17.6	17.6
Ixekizumab	27 (8.6)	61 (11.1)	49 (8.9)	0.025	9.5	9.5	9.5
Meteorological metrics during treatment^d^							
Temperature, °C	19.0 (4.6)	19.1 (8.4)	16.5 (6.3)	0.392	18.1 (4.7)	18.1 (8.6)	18.1 (6.3)
UV index	9.0 (1.3)	7.9 (2.6)	6.1 (2.0)	1.432	7.7 (1.5)	7.7 (2.9)	7.7 (2.1)
Humidity, %	72.9 (4.3)	75.6 (5.5)	75.5 (4.0)	0.566	75.0 (4.3)	75.0 (5.5)	75.0 (3.9)

BMI, body mass index; NAFLD, non-alcoholic fatty liver disease; BMI, body mass index; PASI, Psoriasis Area and Severity Index; PGA, Physician’s Global Assessment; DLQI, Dermatology Quality of Life Index; UV, ultraviolet. Data are provided as mean (standard deviation) unless otherwise stated. ^a^After covariate balancing propensity score (CBPS) weighting, a single individual no longer represents a single data entity and thus raw counts are not reported after weighting. ^b^CBPS weighting provided precise balance of mean values of covariates (maximum absolute standardized difference < 0.001). ^c^The maximum absolute standardized difference observed among all pairwise comparisons. ^d^Mean monthly temperature, UV index, and humidity during the patient’s treatment period.

### Outcomes

Unadjusted and adjusted analyses assessed response rates and ORs for PASI 75, PASI 90, PGA 0/1, and DLQI MID across warming, transition, and cooling groups at 3 months (**Table 2**). Response rates for PASI 75 were 58.0%, 57.3%, and 49.2% for warming, transition, and cooling groups, respectively. Adjusted analyses revealed significantly lower odds for the cooling group compared to the warming group (adjusted OR 0.70, 95% CI 0.61–0.80, *P* <.001), while the transition group showed no significant difference (adjusted OR 0.97, 95% CI 0.84–1.11, *P* = .671). For PASI 90, response rates were 37.4%, 34.7%, and 28.9%, respectively, with the cooling group showing reduced odds (adjusted OR 0.68, 95% CI 0.59–0.79, *P* <.001) compared to the warming group, while the transition group again showed no significant difference (adjusted OR 0.89, 95% CI 0.77–1.02, *P* = .100). For absolute PASI < 3, response rates were 47.2%, 44.1%, and 38.5% in the warming, transition, and cooling groups, respectively. Patients in the cooling group had significantly reduced odds of achieving PASI < 3 (adjusted OR 0.69, 95% CI 0.57–0.76; *P* < .001), whereas the transition group showed no significant difference compared with the warming group (adjusted OR 0.92, 95% CI 0.74–1.01; *P* = .733). PGA 0/1 response rates were 64.3%, 60.4%, and 54.0%, with the cooling group demonstrating significantly lower odds (adjusted OR 0.65, 95% CI 0.57–0.75, *P* <.001) compared to the warming group, and the transition group showing slightly reduced odds (adjusted OR 0.85, 95% CI 0.74–0.98, *P* = .023). For DLQI MID, response rates were 54.2%, 54.7%, and 50.5%, with the cooling group showing significantly lower odds (adjusted OR 0.86, 95% CI 0.75–0.99, *P* = .032), and no significant difference for the transition group (adjusted OR 1.02, 95% CI 0.89–1.17, *P* = .786). After multiplicity adjustment, the results for PASI 75, PASI 90, and PGA 0/1 remained statistically significant (Hommel-adjusted *P* <.001). Additionally, a linear trend was observed across the warming, transition, and cooling groups, with treatment effectiveness progressively decreasing from the warming to the cooling group. Consistent patterns were observed in the 2-month analysis (**Supplementary Table S3**; see **Supplementary Material**).

**Table 2:** Response rates and odds ratios (ORs) for PASI 75, PASI 90, PGA 0/1, and DLQI MID at 3 months post-treatment.

Temperature trend group	Unadjusted analysis			Adjusted analysis^a^				
	Responders, n/N (%)^b^	OR (95% CI)^c^	*P*-value	Responders, % (95% CI)^c^	OR (95% CI)^c^	*P*-value	*P*-value (Hommel-adjusted)	*P* for trend (Hommel-adjusted)
PASI 75								
Warming	181/313 (57.8)	Ref		58.0 (50.5, 65.5)	Ref			
Transition	345/548 (63.0)	1.24 (0.93, 1.65)	.138	57.3 (52.4, 62.2)	0.97 (0.84, 1.11)	.671	1.000	
Cooling	261/550 (47.5)	0.66 (0.50, 0.87)	**.003**	49.2 (42.3, 56.0)	0.70 (0.61, 0.80)	**<.001**	**<.001**	**<.001**
PASI 90								
Warming	112/313 (35.8)	Ref		37.4 (30.1, 44.8)	Ref			
Transition	206/548 (37.6)	1.08 (0.81, 1.44)	.597	34.7 (29.9, 39.4)	0.89 (0.77, 1.02)	.100	.800	
Cooling	146/550 (26.5)	0.65 (0.48, 0.87)	**.004**	28.9 (22.7, 35.1)	0.68 (0.59, 0.79)	**<.001**	**<.001**	**<.001**
PGA 0/1								
Warming	190/312 (60.9)	Ref		64.3 (57.0, 71.6)	Ref			
Transition	340/545 (62.4)	1.05 (0.79, 1.40)	.727	60.4 (55.6, 65.3)	0.85 (0.74, 0.98)	**.023**	.276	
Cooling	305/549 (55.6)	0.80 (0.60, 1.05)	.112	54.0 (47.2, 60.8)	0.65 (0.57, 0.75)	**<.001**	**<.001**	**<.001**
DLQI MID								
Warming	147/272 (54.0)	Ref		54.2 (46.6, 61.8)	Ref			
Transition	263/459 (57.3)	1.15 (0.87, 1.52)	.326	54.7 (49.8, 59.6)	1.02 (0.89, 1.17)	.786	1.000	
Cooling	232/484 (47.9)	0.75 (0.56, 0.99)	**.039**	50.5 (43.6, 57.3)	0.86 (0.75, 0.99)	**.032**	.352	.352

PASI, Psoriasis Area and Severity Index; PGA, Physician’s Global Assessment; DLQI MID, Dermatology Quality of Life Index minimal important difference; CI, confidence interval. ^a^Balancing the exposure groups using covariate balancing propensity score (CBPS) weighting, adjusted for confounders including age, sex, BMI, disease duration, prior use of biologic and non-biologic systemic therapies, phototherapy, smoking status, family history of psoriasis, psoriatic arthritis, comorbidities (cardiovascular disease, diabetes, NAFLD, hypertension, hyperlipidemia, and hyperuricemia), baseline PASI, PGA, and DLQI scores, as well as mean temperature and humidity during treatment. ^b^As-observed results. ^c^Multiple imputation results. Significant *P*-values are highlighted in bold (*P* < 0.05).

RDs and NNTs were calculated to assess the impact of temperature trend-defined seasonality on achieving PASI 75, PASI 90, PGA 0/1, and DLQI MID at 3 and 2 months, offering insights into their clinical significance (**Table 3**, **Supplementary Table S4**; see **Supplementary Material**). For example, for PASI 75 at 3 months, the adjusted NNT is -11.4, meaning that treating about 11 patients in the warming group would lead to 1 additional patient achieving PASI 75 at 3 months compared to the cooling group.

**Table 3:** Risk differences (RDs) and numbers needed to treat (NNTs) for PASI 75, PASI 90, PGA 0/1, and DLQI MID at 3 months post-treatment.

Temperature trend group	Unadjusted analysis			Adjusted analysis^a^		
	RD (95% CI)^b^	*P*-value	NNT^c^	RD (95% CI)^b^	*P*-value	NNT^c^
PASI 75						
Warming	Ref					
Transition	0.051 (-0.017, 0.120)	.142	19.6	-0.007 (-0.071, 0.056)	.821	-142.9
Cooling	-0.104 (-0.172, -0.035)	**.003**	-9.6 (-28.6, -5.8)	-0.088 (-0.152, -0.025)	**.006**	-11.4 (-40.0, -6.6)
PASI 90						
Warming	Ref					
Transition	0.018 (-0.047, 0.083)	.585	55.6	-0.028 (-0.088, 0.033)	.368	-35.7
Cooling	-0.092 (-0.157, -0.027)	**.005**	-10.9 (-37.0, -6.4)	-0.085 (-0.145, -0.025)	**.006**	-11.8 (-40.0, -6.9)
PGA 0/1						
Warming	Ref					
Transition	0.012 (-0.056, 0.080)	.729	83.3	-0.039 (-0.101, 0.024)	.226	-25.6
Cooling	-0.056 (-0.124, 0.012)	.109	-17.9	-0.103 (-0.165, -0.040)	**.001**	-9.7 (-25.0, -6.1)
DLQI MID						
Warming	Ref					
Transition	0.034 (-0.035, 0.103)	.328	29.4	0.005 (-0.059, 0.069)	.884	200.0
Cooling	-0.073 (-0.142, -0.004)	**.038**	-13.7 (-250.0, -7.0)	-0.037 (-0.101, 0.026)	.250	-27.0

PASI, Psoriasis Area and Severity Index; PGA, Physician’s Global Assessment; DLQI MID, Dermatology Quality of Life Index minimal important difference; CI, confidence interval. ^a^Balancing the exposure groups using covariate balancing propensity score (CBPS) weighting, adjusted for confounders including age, sex, BMI, disease duration, prior use of biologic and non-biologic systemic therapies, phototherapy, smoking status, family history of psoriasis, psoriatic arthritis, comorbidities (cardiovascular disease, diabetes, NAFLD, hypertension, hyperlipidemia, and hyperuricemia), baseline PASI, PGA, and DLQI scores, as well as mean temperature and humidity during treatment. ^b^Multiple imputation results. ^c^95% CIs are only shown for NNTs where the corresponding RD CI did not contain 0. Significant *P*-values are highlighted in bold (*P* < 0.05).

### Sensitivity analyses

A series of sensitivity analyses were conducted to validate the robustness of findings for the 3-month analysis (**Table 4**). Across all analyses, the cooling group consistently exhibited significantly lower odds of achieving PASI 75 (ORs 0.60–0.75), PASI 90 (ORs 0.63–0.78), and PGA 0/1 (ORs 0.58–0.82) compared to the warming group. For DLQI MID, odds were also reduced but showed less consistency, with some analyses yielding marginal significance (e.g., OR 0.75–0.88). The transition group demonstrated intermediate effects, with no significant differences observed in most scenarios. Similar results were observed in the 2-month analysis (**Supplementary Table S5**; see **Supplementary Material**).

**Table 4:** Effectiveness in the sensitivity analyses at 3 months post-treatment.

Temperature trend group	Sensitivity analysis 1^a^		Sensitivity analysis 2^b^		Sensitivity analysis 3^c^		Sensitivity analysis 4^d^		Sensitivity analysis 5^e^	
	Adjusted OR (95% CI)	*P*-value	Adjusted OR (95% CI)	*P*-value	Adjusted OR (95% CI)	*P*-value	Adjusted OR (95% CI)	*P*-value	Adjusted OR (95% CI)	*P*-value
PASI 75										
Warming	Ref		Ref		Ref		Ref		Ref	
Transition	0.87 (0.75, 1.01)	.076	1.24 (0.93, 1.65)	.138	0.96 (0.81, 1.13)	.602	0.93 (0.80, 1.08)	.602	0.99 (0.86, 1.15)	.908
Cooling	0.60 (0.52, 0.70)	**<.001**	0.66 (0.50, 0.87)	**.003**	0.75 (0.64, 0.89)	**.001**	0.72 (0.62, 0.83)	**<.001**	0.72 (0.62, 0.83)	**<.001**
PASI 90										
Warming	Ref		Ref		Ref		Ref		Ref	
Transition	0.80 (0.69, 0.94)	**.005**	1.08 (0.81, 1.44)	.597	0.92 (0.78, 1.09)	.345	0.85 (0.73, 0.99)	.041	0.83 (0.71, 0.96)	**.012**
Cooling	0.63 (0.54, 0.73)	**<.001**	0.65 (0.48, 0.87)	**.004**	0.78 (0.65, 0.93)	**.004**	0.71 (0.60, 0.83)	**<.001**	0.63 (0.54, 0.73)	**<.001**
PGA 0/1										
Warming	Ref		Ref		Ref		Ref		Ref	
Transition	0.83 (0.72, 0.97)	.018	0.88 (0.76, 1.03)	.115	0.73 (0.61, 0.87)	<.001	0.78 (0.67, 0.91)	**.002**	0.85 (0.73, 0.99)	**.037**
Cooling	0.58 (0.50, 0.68)	**<.001**	0.82 (0.70, 0.96)	**.013**	0.66 (0.56, 0.79)	**<.001**	0.64 (0.55, 0.75)	**<.001**	0.65 (0.56, 0.75)	**<.001**
DLQI MID										
Warming	Ref		Ref		Ref		Ref		Ref	
Transition	0.86 (0.74, 1.00)	.050	1.15 (0.87, 1.52)	.326	1.15 (0.97, 1.36)	.108	1.00 (0.86, 1.16)	.973	1.03 (0.90, 1.20)	.647
Cooling	0.85 (0.73, 0.98)	**.027**	0.75 (0.56, 0.99)	**.039**	0.77 (0.65, 0.91)	**.002**	0.86 (0.74, 1.00)	**.048**	0.88 (0.77, 1.02)	.094

OR, odds ratio; CI, confidence interval; PASI, Psoriasis Area and Severity Index; PGA, Physician’s Global Assessment; DLQI MID, Dermatology Quality of Life Index minimal important difference. ^a^Sensitivity analysis 1: Replacing mean temperature with mean UV index during treatment in the CBPS model. ^b^Sensitivity analysis 2: Truncating CBPS weights at the 5^th^ and 95^th^. ^c^Sensitivity analysis 3: Excluding patients undergoing phototherapy. ^d^Sensitivity analysis 4: Excluding patients exposed to mean temperatures ≤ 10^th^ percentile. ^e^Sensitivity analysis 5: Excluding patients with baseline PASI scores in the ≥ 90^th^ percentile.

### E-value

**Figure 3:**
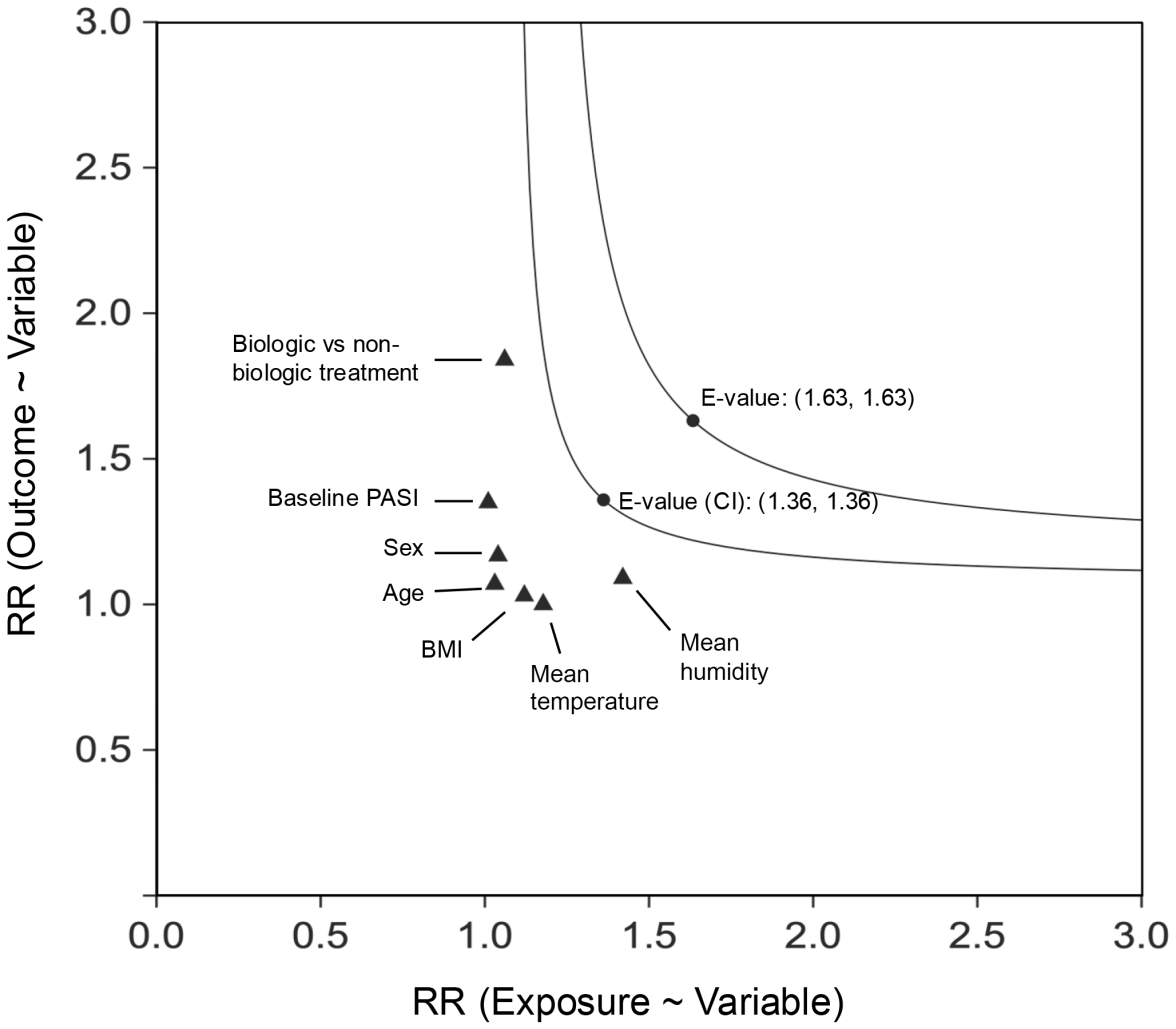
E-Value for PASI 75 at 3 months (multiple imputation), the cooling group vs. the warming group. CI, confidence interval; RR, relative risk. The E-value was calculated based on the RR for the cooling group compared to the warming group, with a separate E-value derived from the upper limit of the RR’s confidence interval. The x-axis represents the extent of imbalance in the prevalence of the unmeasured confounder between the two exposure cohorts, while the y-axis denotes the strength of the association between the unmeasured confounder and the outcome (PASI 75 at 3 months). For comparison, the effects of established confounders, with continuous variables dichotomized at the median, have been included.

The E-value for PASI 75 at 3 months comparing the cooling group vs. the warming group was 1.63, with an E-value of 1.36 for the upper confidence limit of the point estimate. These values, when compared to the risk ratios of known factors such as sex, age, BMI, baseline PASI, treatment type, mean temperature, and humidity during treatment, suggest it is highly unlikely that an unmeasured confounder could fully negate the observed association (**Figure 3**). Similar findings were observed in the 2-month analysis (**Supplementary Figure S1**; see **Supplementary Material**).

### Interaction analyses

Interaction analyses assessed differential effects of temperature trends (cooling group vs warming group) on PASI 75 attainment at 3 and 2 months. In the 3−month analysis, most predictors showed no significant interactions. BMI demonstrated a significant interaction on both the multiplicative scale (OR 0.45, 95% CI 0.24–0.86, *P* = .016) and additive scale (RD -0.150, 95% CI -0.275 to -0.025, *P* = .019), indicating worse responses to cooling trends in patients with higher BMI (**Table 5**). Subgroup analyses showed that the estimated OR for cooling vs warming was 0.42 (95% CI 0.26–0.70) in the high−BMI group (BMI ≥ 24.5) and 1.26 (95% CI 0.71–1.99) in the low−BMI group (BMI < 24.5), highlighting that the seasonal effect was most pronounced in high−BMI patients. Interestingly, treatment type did not modify the seasonal effect on PASI 75 outcomes. Similar findings were observed in the 2−month analysis (**Supplementary Table S6**; see **Supplementary Material**).

**Table 5:** Interaction analyses of temperature trends (the cooling group vs. the warming group) with predictors for PASI 75 at 3 months.

Predictor^c^	Interaction of ORs^a^		Interaction of RDs^b^	
	Interaction (95% CI)	*P*-value	Interaction (95% CI)	*P*-value
Age (49 years)	1.53 (0.81, 2.91)	.193	0.080 (-0.045, 0.204)	.212
Male sex	1.18 (0.62, 2.24)	.970	-0.002 (-0.148, 0.144)	.978
Disease duration (11 years)	1.18 (0.62, 2.24)	.607	0.028 (-0.097, 0.152)	.662
BMI (24.5)	0.45 (0.24, 0.86)	**.016**	-0.150 (-0.275, -0.025)	**.019**
Baseline PASI (12.0)	0.87 (0.45, 1.67)	.677	-0.020 (-0.146, 0.106)	.754
Baseline DLQI (9.0)	1.38 (0.72, 2.63)	.326	0.064 (-0.061, 0.190)	.316
Baseline PGA 3, 4 vs. 1, 2	1.14 (0.58, 2.23)	.703	0.027 (-0.105, 0.159)	.685
Mean temperature during treatment (19 °C)	1.27 (0.65, 2.46)	.483	0.048 (-0.082, 0.177)	.470
Mean humidity during treatment (73.4%)	0.82 (0.43, 1.58)	.556	-0.036 (-0.164, 0.092)	.577
Mean UV index during treatment (7.8)	1.51 (0.57, 4.03)	.410	0.087 (-0.101, 0.276)	.363
Treatment				
Methotrexate	1.15 (0.37, 3.55)	.812	0.009 (-0.211, 0.228)	.939
Phototherapy	0.95 (0.31, 2.92)	.927	-0.033(-0.252, 0.185)	.766
Adalimumab	1.24 (0.16, 9.41)	.833	0.041 (-0.387, 0.469)	.850
Ustekinumab	0.87 (0.18, 4.14)	.865	-0.049 (-0.359, 0.260)	.756
Guselkumab	2.89 (0.24, 5.11)	.187	0.158 (-0.230, 0.546)	.425
Secukinumab	0.66 (0.15, 2.87)	.583	-0.049 (-0.296, 0.198)	.696
Ixekizumab	0.88 (0.16, 4.89)	.879	0.008 (-0.267, 0.284)	.953
Smoker	1.37 (0.73, 2.59)	.332	0.054 (-0.070, 0.179)	.392
Family history	1.04 (0.49, 2.18)	.925	0.004 (-0.141, 0.149)	.956
College education	0.73 (0.38, 1.38)	.326	-0.052 (-0.177, 0.072)	.410
Prior biologics	1.33 (0.46, 3.85)	.597	0.050 (-0.153, 0.253)	.629
Prior non-biologics	1.14 (0.58, 2.24)	.701	0.027 (-0.104, 0.158)	.687
Prior phototherapy	1.37 (0.72, 2.62)	.336	0.061 (-0.065, 0.187)	.344
Exacerbation season				
Spring	1.60 (0.61, 4.20)	.342	0.089 (-0.103, 0.281)	.362
Summer	0.48 (0.11, 2.16)	.342	-0.146 (-0.429, 0.137)	.312
Autumn	1.40 (0.56–3.51)	.467	0.064 (-0.116, 0.245)	.485
Winter	1.33 (0.69, 2.57)	.394	0.058 (-0.071, 0.187)	.379
Psoriatic arthritis	2.47 (0.81, 7.52)	.111	0.177 (-0.042, 0.396)	.114
Comorbidities				
Cardiovascular disease	0.53 (0.13, 2.18)	.375	-0.145 (-0.421, 0.131)	.304
Diabetes	2.91 (0.93, 7.50)	.126	0.209 (-0.023, 0.395)	.228
Hypertension	1.43 (0.71, 2.91)	.319	0.068 (-0.070, 0.207)	.334
NAFLD	0.45 (0.16, 1.24)	.122	-0.160 (-0.355, 0.035)	.109
Hyperlipidemia	1.91 (0.75, 4.86)	.175	0.124 (-0.064, 0.312)	.195
Hyperuricemia	1.33 (0.43–4.09)	.621	0.067 (-0.160, 0.293)	.566

PASI, Psoriasis Area and Severity Index; OR, odds ratio; RD, risk difference; CI, confidence interval; BMI, body mass index; PGA, Physician’s Global Assessment; DLQI MID, Dermatology Quality of Life Index minimal important difference; UV, ultraviolet; NAFLD, non-alcoholic fatty liver disease. ^a^Defined as the ratio of ORs which compares the ORs in patients in the cooling group vs. the warming group across the specified subgroups. ^b^Defined as the difference in RDs, which is the absolute difference in RDs in patients in the cooling group vs. the warming group across the specified subgroups. ^c^Continuous variables are dichotomized at the median. Significant *P*-values are highlighted in bold (*P* < 0.05).

## Discussion

This study highlights the impact of temperature trend-defined seasonality on the effectiveness of systemic therapies for psoriasis. Cooling trends are linked to lower odds of achieving PASI 75, PASI 90, and PGA 0/1, while the effect on DLQI MID is weaker and less consistent. This suggests that seasonal influences primarily impair clinical response, with only a modest impact on patient-reported quality of life. Clinically relevant associations are quantified using RDs and NNTs. Interaction analyses reveal that BMI significantly influences outcomes, with higher BMI patients responding less favorably to cooling trends than those with lower BMI.

It is widely believed that psoriasis exhibits a pronounced seasonal pattern, influencing lesion severity, symptoms, flare-ups and remissions, as well as treatment initiation and discontinuation (20–24). While many studies report that skin lesions in psoriasis patients improve during warmer seasons, this observation is not consistently supported across all research (12, 25). To achieve more consistent research outcomes, it is essential to clearly define the primary exposure, seasonality. In prior studies, two commonly recognized methods have been used to define seasons (8). The first, based on astronomical criteria, designates spring as March 22-June 21, summer as June 22-September 21, autumn as September 22-December 21, and winter as December 22-March 21. The second, based on meteorological criteria, defines spring as March 1-May 31, summer as June 1-August 31, autumn as September 1-November 30, and winter as December 1-February 28. Both methods have notable limitations. Astronomical definitions rely on fixed dates misaligned with climatic changes and lack adaptability to regional variations, especially in tropical and subtropical areas. Meteorological definitions, dividing the year into fixed three-month intervals, ignore real-world climate variability and gradual environmental transitions. In contrast, temperature-trend defined seasonality aligns with actual climatic changes, accommodates regional differences, and captures transitional periods, offering a more dynamic and nuanced understanding of environmental impacts.

Previous studies have shown that sunlight, UV radiation, temperature, and humidity influence the onset, severity, and treatment outcomes of psoriasis through various immune mechanisms (26–31). In our findings, temperature trend-defined seasonality impacts treatment outcomes independently of average levels of temperature, UV, and humidity. While this does not rule out the role of static meteorological factors, we identified a novel influence—meteorological trends, exemplified by temperature trends, which may act as a “stressor” that modifies the intrinsic disease activity in patients. This observation aligns with the concept of dynamic psoriasis disease activity proposed by Mrowietz et al., emphasizing the role of environmental triggers such as temperature variation (32). The Mrowietz’s study reported that approximately 30% of patients had “winter−type” psoriasis, suggesting that seasonal effects may be most pronounced in certain subgroups. In our Shanghai cohort, cooling trends reduced treatment response at the group level but were most evident in high−BMI patients, reflecting variable individual susceptibility. Overall, these findings are consistent with ACTIPSO, although the magnitude of seasonal effects may differ according to patient characteristics.

This study’s strengths include leveraging a large, multicentre cohort with comprehensive patient and environmental data, enabling robust evaluation of seasonal effects on treatment outcomes. Methodologically, the application of CBPS weighting effectively minimized baseline confounding, ensuring that observed associations reliably reflected causal relationships. Additionally, using interaction analyses, the research identifies specific subgroups, such as those defined by BMI, that experience differential responses to seasonal variations. These findings emphasize the importance of considering individual patient characteristics when evaluating therapeutic responses. Clinically interpretable effect sizes, including RDs and NNTs, further enhanced the practical applicability of the findings.

These findings have important implications for clinical study design and reporting. Seasonal effects may act as confounders, especially in trials of biologic efficacy or dose reduction. To enhance validity, future studies should report environmental conditions (temperature trends, UV exposure, humidity) and apply seasonal stratification or balancing. In real−world studies, sensitivity analyses can help distinguish true drug effects from seasonal influences.

These findings highlight the clinical importance of considering seasonality in psoriasis management. Cooling seasons may require more proactive, individualized strategies, particularly for high−BMI patients, who showed up to a 15% lower PASI 75 response rate. For these patients, clinicians should avoid dose tapering or interval extension, ensure closer monitoring, and consider dose optimization or early combination therapy if responses are suboptimal. Incorporating seasonal awareness with patient−specific risk factors supports a personalized approach. Moreover, future clinical trials should account for temperature trend−defined seasonality as a potential confounder and use participant stratification or balancing to ensure validity across different climate zones.

Several limitations should be acknowledged. First, the study’s observational design precludes definitive causal inferences. Although we applied robust CBPS to minimize baseline confounding, unmeasured factors—such as patient stress, therapy adherence, or physician prescribing habits—may have influenced treatment selection and outcomes. For example, the higher proportion of biologics in the warming period and the greater use of acitretin in the cooling period likely reflect complex clinical decision-making influenced by seasonality, patient characteristics, and drug availability. Second, reliance on regional climate data may not fully capture individual exposure, especially for patients living in microclimates or spending substantial time indoors. Third, while the results suggest a mechanistic link between seasonality and therapeutic outcomes, direct biological evidence (e.g., inflammatory biomarkers or skin barrier function) was not assessed. Future studies will incorporate longitudinal biological data, including serum inflammatory markers and skin transcriptomics, to better elucidate the immunologic and molecular basis of seasonal effects. Fourth, our findings from Shanghai’s subtropical monsoon climate may not generalize to other regions, as seasonal effects could be weaker in tropical areas, amplified in arid climates, or more pronounced in continental and polar regions. Multicenter studies across diverse climates are needed to validate and refine region-specific recommendations. Finally, the focus on short-term outcomes (2–3 months) limits the scope of this study; longer follow-up may reduce seasonal disparities due to increasing exposure to transitional conditions between warming and cooling periods.

In conclusion, this study highlights the significant impact of temperature trend-defined seasonality on psoriasis treatment outcomes, with cooling trends linked to reduced effectiveness. By introducing a novel seasonality framework based on temperature variation, we offer fresh insights into the environmental factors shaping therapeutic responses. The observed BMI-modified effect underscores the importance of developing tailored management strategies that integrate environmental factors with individual patient characteristics.

## Data Availability

The original contributions presented in the study are included in the article/**Supplementary Material**. Further inquiries can be directed to the corresponding author.

## Supplementary material

Supplementary Figure 1 E-Value for PASI 75 at 2 months (multiple imputation), the cooling group vs. the warming group. CI, confidence interval; RR, relative risk. The E-value was calculated based on the RR for the cooling group compared to the warming group, with a separate E-value derived from the upper limit of the RR’s confidence interval. The x-axis represents the extent of imbalance in the prevalence of the unmeasured confounder between the two exposure cohorts, while the y-axis denotes the strength of the association between the unmeasured confounder and the outcome (PASI 75 at 2 months). For comparison, the effects of established confounders, with continuous variables dichotomized at the median, have been included.

## References

[1] Griffiths CEM Armstrong AW Gudjonsson JE Barker J. Psoriasis. Lancet (London England). (2021) 397:1301–15. doi: 10.1016/s0140-6736(20)32549-6, PMID: 33812489

[2] Dand N Mahil SK Capon F Smith CH Simpson MA Barker JN. Psoriasis and genetics. Acta dermato-venereologica. (2020) 100:adv00030. doi: 10.2340/00015555-3384, PMID: 31971603 PMC9128944

[3] Dika E Bardazzi F Balestri R Maibach HI. Environmental factors and psoriasis. Curr problems Dermatol. (2007) 35:118–35. doi: 10.1159/000106419, PMID: 17641494

[4] Teng Y Xie W Tao X Liu N Yu Y Huang Y. Infection-provoked psoriasis: induced or aggravated (Review). Exp Ther Med. (2021) 21:567. doi: 10.3892/etm.2021.9999, PMID: 33850539 PMC8027725

[5] Madden SK Flanagan KL Jones G. How lifestyle factors and their associated pathogenetic mechanisms impact psoriasis. Clin Nutr (Edinburgh Scotland). (2020) 39:1026–40. doi: 10.1016/j.clnu.2019.05.006, PMID: 31155371

[6] Balak DM Hajdarbegovic E. Drug-induced psoriasis: clinical perspectives. Psoriasis (Auckland NZ). (2017) 7:87–94. doi: 10.2147/ptt.S126727, PMID: 29387611 PMC5774610

[7] Jensen KK Serup J Alsing KK. Psoriasis and seasonal variation: A systematic review on reports from northern and central europe-little overall variation but distinctive subsets with improvement in summer or wintertime. Skin Res technology: Off J Int Soc Bioengineering Skin (ISBS) [and] Int Soc Digital Imaging Skin (ISDIS) [and] Int Soc Skin Imaging (ISSI). (2022) 28:180–6. doi: 10.1111/srt.13102, PMID: 34758175 PMC9907615

[8] Hancox JG Sheridan SC Feldman SR Fleischer AB Jr. Seasonal variation of dermatologic disease in the USA: A study of office visits from 1990 to 1998. Int J Dermatol. (2004) 43:6–11. doi: 10.1111/j.1365-4632.2004.01828.x, PMID: 14693014

[9] Pascoe VL Kimball AB. Seasonal variation of acne and psoriasis: A 3-year study using the physician global assessment severity scale. J Am Acad Dermatol. (2015) 73:523–5. doi: 10.1016/j.jaad.2015.06.001, PMID: 26282801

[10] Muddasani S Fleischer AB Jr. Common skin diseases reveal seasonal variation in internet search interest. Skinmed. (2022) 20:233–4., PMID: 35779034

[11] Brito LAR Nascimento A Marque C Miot HA. Seasonality of the hospitalizations at a dermatologic ward (2007-2017). Anais brasileiros dermatologia. (2018) 93:755–8. doi: 10.1590/abd1806-4841.20187309, PMID: 30156635 PMC6106666

[12] Kubota K Kamijima Y Sato T Ooba N Koide D Iizuka H. Epidemiology of psoriasis and palmoplantar pustulosis: A nationwide study using the Japanese national claims database. BMJ Open. (2015) 5:e006450. doi: 10.1136/bmjopen-2014-006450, PMID: 25588781 PMC4298108

[13] Yu N Peng C Zhou J Gu J Xu J Li X. Measurement properties of the patient global assessment numerical rating scale in moderate-to-severe psoriasis. Br J Dermatol. (2023) 189:437–46. doi: 10.1093/bjd/ljad188, PMID: 37310289

[14] Imai K Ratkovic M. Covariate balancing propensity score. J R Stat Soc Ser B-Statistical Method. (2014) 76:243–63. doi: 10.1111/rssb.12027

[15] Fan JQ Imai K Lee I Liu H Ning Y Yang XL. Optimal covariate balancing conditions in propensity score estimation. J Business Economic Stat. (2022) 41:97–110. doi: 10.1080/07350015.2021.2002159

[16] Laupacis A Sackett DL Roberts RS. An assessment of clinically useful measures of the consequences of treatment. New Engl J Med. (1988) 318:1728–33. doi: 10.1056/nejm198806303182605, PMID: 3374545

[17] VanderWeele TJ Ding P. Sensitivity analysis in observational research: introducing the E-value. Ann Internal Med. (2017) 167:268–74. doi: 10.7326/m16-2607, PMID: 28693043

[18] Austin PC White IR Lee DS van Buuren S. Missing data in clinical research: A tutorial on multiple imputation. Can J Cardiol. (2021) 37:1322–31. doi: 10.1016/j.cjca.2020.11.010, PMID: 33276049 PMC8499698

[19] Hommel G. A stagewise rejective multiple test procedure based on a modified bonferroni test. Biometrika. (1988) 75:383–6. doi: 10.2307/2336190

[20] Watad A Azrielant S Bragazzi NL Sharif K David P Katz I. Seasonality and autoimmune diseases: the contribution of the four seasons to the mosaic of autoimmunity. J Autoimmun. (2017) 82:13–30. doi: 10.1016/j.jaut.2017.06.001, PMID: 28624334

[21] Bedi TR. Psoriasis in north India. Geographical Variations. Dermatologica. (1977) 155:310–4. doi: 10.1159/000250983, PMID: 902843

[22] Ferguson FJ Lada G Hunter HJA Bundy C Henry AL Griffiths CEM. Diurnal and seasonal variation in psoriasis symptoms. J Eur Acad Dermatol Venereology: JEADV. (2021) 35:e45–e7. doi: 10.1111/jdv.16791, PMID: 32594573

[23] Park BS Youn JI. Factors influencing psoriasis: an analysis based upon the extent of involvement and clinical type. J Dermatol. (1998) 25:97–102. doi: 10.1111/j.1346-8138.1998.tb02357.x, PMID: 9563276

[24] Niedźwiedź M Skibińska M Ciążyńska M Noweta M Czerwińska A Krzyścin J. Psoriasis and seasonality: exploring the genetic and epigenetic interactions. Int J Mol Sci. (2024) 25:11670. doi: 10.3390/ijms252111670, PMID: 39519223 PMC11547062

[25] Harvell JD Selig DJ. Seasonal variations in dermatologic and dermatopathologic diagnoses: A retrospective 15-year analysis of dermatopathologic data. Int J Dermatol. (2016) 55:1115–8. doi: 10.1111/ijd.13229, PMID: 27061329

[26] Green C Diffey BL Hawk JL. Ultraviolet radiation in the treatment of skin disease. Phys Med Biol. (1992) 37:1–20. doi: 10.1088/0031-9155/37/1/001, PMID: 1741417

[27] Bulat V Situm M Dediol I Ljubicić I Bradić L. The mechanisms of action of phototherapy in the treatment of the most common dermatoses. Collegium antropologicum. (2011) 35 Suppl 2:147–51., PMID: 22220423

[28] Jacobson CC Kumar S Kimball AB. Latitude and psoriasis prevalence. J Am Acad Dermatol. (2011) 65:870–3. doi: 10.1016/j.jaad.2009.05.047, PMID: 21920244

[29] Wan MJ Su XY Zheng Y Gong ZJ Yi JL Zhao Y. Seasonal variability in the biophysical properties of forehead skin in women in guangzhou city, China. Int J Dermatol. (2015) 54:1319–24. doi: 10.1111/ijd.12741, PMID: 25557023

[30] Egawa M Tagami H. Comparison of the depth profiles of water and water-binding substances in the stratum corneum determined *in vivo* by raman spectroscopy between the cheek and volar forearm skin: effects of age, seasonal changes and artificial forced hydration. Br J Dermatol. (2008) 158:251–60. doi: 10.1111/j.1365-2133.2007.08311.x, PMID: 18047517

[31] Shinohara K Hara-Chikuma M. Low humidity altered the gene expression profile of keratinocytes in a three-dimensional skin model. Mol Biol Rep. (2022) 49:7465–74. doi: 10.1007/s11033-022-07549-0, PMID: 35579735

[32] Mrowietz U Dieckmann T Gerdes S Szymczak S von Spreckelsen R Körber A. Actipso: definition of activity types for psoriatic disease: A novel marker for an advanced disease classification. J Eur Acad Dermatol Venereology: JEADV. (2021) 35:2027–33. doi: 10.1111/jdv.17434, PMID: 34076926

