# Supplementary figures and images for: Impact of temperature trend-defined seasonality on psoriasis treatment outcomes: a multicenter longitudinal study
Source: Front Immunol. 2025 Sep 17;16:1641225. doi: 10.3389/fimmu.2025.1641225 (PMC12484154; doi:10.3389/fimmu.2025.1641225)

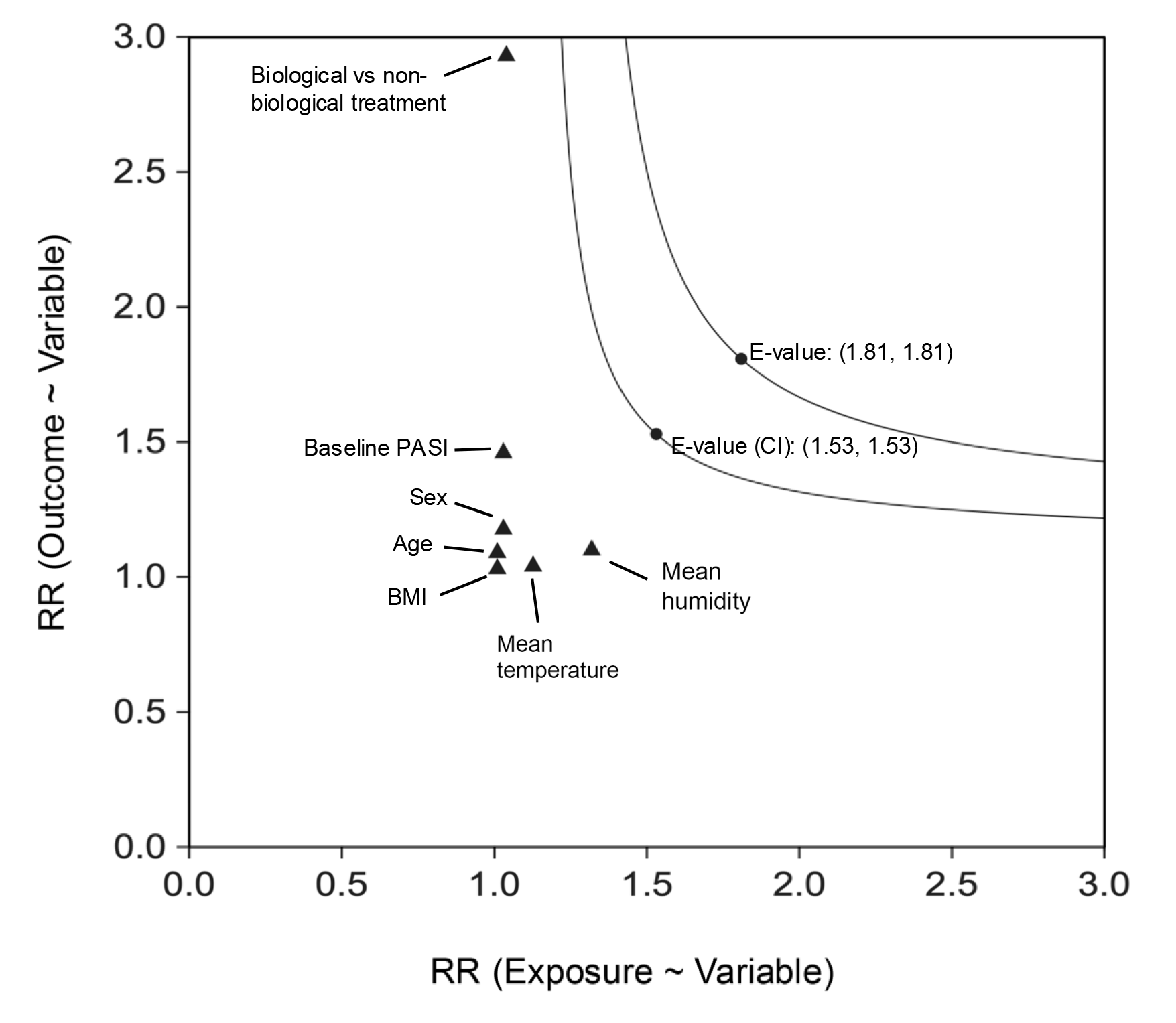

Supplement: Supplementary Figure 1 — E-Value for PASI 75 at 2 months (multiple imputation), the cooling group vs. the warming group. CI, confidence interval; RR, relative risk. The E-value was calculated based on the RR for the cooling group compared to the warming group, with a separate E-value derived from the upper limit of the RR’s confidence interval. The x-axis represents the extent of imbalance in the prevalence of the unmeasured confounder between the two exposure cohorts, while the y-axis denotes the strength of the association between the unmeasured confounder and the outcome (PASI 75 at 2 months). For comparison, the effects of established confounders, with continuous variables dichotomized at the median, have been included. [file Image1.tif]
