# Supplementary material for: Impact of temperature trend-defined seasonality on psoriasis treatment outcomes: a multicenter longitudinal study
Source: Front Immunol. 2025 Sep 17;16:1641225. doi: 10.3389/fimmu.2025.1641225 (PMC12484154; doi:10.3389/fimmu.2025.1641225)
Supplement: Supplementary file 2 [file Table1.docx]

**Table S1** Patient demographic and clinical characteristics at baseline in the 2-month analysis

|  | **Unweighted population** | | | | **Weighted population^a,b^** | | |
| --- | --- | --- | --- | --- | --- | --- | --- |
|  | **Warming** | **Transition** | **Cooling** | **Absolute standardized difference^c^** | **Warming** | **Transition** | **Cooling** |
| Total, n | 391 | 272 | 681 |  |  |  |  |
| Age, years | 48.8 (15.7) | 47.5 (15.4) | 49.8 (15.4) | 0.151 | 49.2 (15.6) | 49.3 (15.3) | 49.2 (15.8) |
| Male sex, n (%) | 291 (74.4) | 195 (71.7) | 516 (75.8) | 0.041 | 75.2 | 75.1 | 75.1 |
| BMI, kg/m^2^ | 24.5 (3.4) | 24.7 (3.6) | 24.7 (3.8) | 0.052 | 24.6 (3.2) | 24.6 (3.6) | 24.6 (3.8) |
| College education, n (%) | 199 (50.9) | 161 (59.2) | 320 (47.0) | 0.142 | 52.0 | 51.8 | 51.9 |
| Smoker, ex and current, n (%) | 175 (44.8) | 100 (36.8) | 335 (49.2) | 0.124 | 45.7 | 45.8 | 45.8 |
| Disease duration, years | 14.9 (12.3) | 13.7 (11.0) | 14.8 (12.3) | 0.097 | 14.7 (12.4) | 14.8 (11.8) | 14.8 (12.3) |
| Family history, n (%) | 95 (24.3) | 52 (19.1) | 157 (23.1) | 0.052 | 22.4 | 22.4 | 22.5 |
| Psoriatic arthritis, n (%) | 26 (6.6) | 29 (10.7) | 72 (10.6) | 0.040 | 9.5 | 9.5 | 9.5 |
| Comorbidities, n (%) |  |  |  |  |  |  |  |
| Cardiovascular disease | 21 (5.4) | 11 (4.0) | 34 (5.0) | 0.013 | 5.0 | 5.1 | 5.0 |
| Diabetes | 48 (12.3) | 22 (8.1) | 89 (13.1) | 0.050 | 11.8 | 11.8 | 11.9 |
| Hypertension | 100 (25.6) | 56 (20.6) | 178 (26.1) | 0.056 | 24.7 | 24.8 | 24.8 |
| NAFLD | 41 (10.5) | 26 (9.6) | 66 (9.7) | 0.009 | 9.8 | 9.9 | 9.9 |
| Hyperlipidemia | 31 (7.9) | 23 (8.5) | 77 (11.3) | 0.037 | 9.3 | 9.3 | 9.4 |
| Hyperuricemia | 23 (5.9) | 13 (4.8) | 54 (7.9) | 0.033 | 6.7 | 6.6 | 6.6 |
| Prior treatment, n (%) |  |  |  |  |  |  |  |
| Biologics | 29 (7.4) | 14 (5.1) | 46 (6.8) | 0.019 | 7.2 | 7.0 | 7.2 |
| Systemic nonbiologics | 119 (30.4) | 91 (33.5) | 243 (35.7) | 0.055 | 34.7 | 34.7 | 34.8 |
| Phototherapy | 156 (39.9) | 105 (38.6) | 278 (40.8) | 0.019 | 41.2 | 41.4 | 41.3 |
| Exacerbation season, n (%) |  |  |  |  |  |  |  |
| Spring | 48 (12.3) | 26 (9.6) | 64 (9.4) | 0.029 | 10.4 | 10.3 | 10.3 |
| Summer | 19 (4.9) | 15 (5.5) | 59 (8.7) | 0.038 | 7.1 | 7.2 | 7.1 |
| Autumn | 40 (10.2) | 54 (19.9) | 150 (22.0) | 0.118 | 17.6 | 17.6 | 17.5 |
| Winter | 237 (60.6) | 166 (61.0) | 390 (57.3) | 0.038 | 58.5 | 58.3 | 58.5 |
| Baseline PASI | 13.7 (8.1) | 14.4 (8.3) | 13.9 (7.8) | 0.085 | 14.1 (8.6) | 14.1 (8.3) | 14.1 (7.8) |
| Baseline PGA, n (%) |  |  |  |  |  |  |  |
| 1 = Minimal | 12 (3.1) | 11 (4.0) | 35 (5.1) | 0.021 | 5.0 | 5.0 | 5.0 |
| 2 = Mild | 152 (38.9) | 96 (35.3) | 286 (42.0) | 0.067 | 38.1 | 38.1 | 38.2 |
| 3 = Moderate | 166 (42.5) | 140 (51.5) | 290 (42.6) | 0.090 | 45.2 | 45.2 | 45.0 |
| 4 = Severe | 61 (15.6) | 25 (9.2) | 70 (10.3) | 0.064 | 11.7 | 11.7 | 11.8 |
| Baseline DLQI | 9.4 (6.5) | 11.0 (7.0) | 9.1 (6.8) | 0.260 | 9.6 (6.4) | 9.6 (6.5) | 9.6 (6.9) |
| Treatment, n (%) |  |  |  |  |  |  |  |
| Acitretin | 50 (12.8) | 33 (12.1) | 117 (17.2) | 0.051 | 14.7 | 14.8 | 14.7 |
| Methotrexate | 98 (25.1) | 60 (22.1) | 191 (28.0) | 0.060 | 26.5 | 26.7 | 26.6 |
| Phototherapy | 119 (30.4) | 42 (15.4) | 172 (25.3) | 0.150 | 23.4 | 23.6 | 23.4 |
| Adalimumab | 7 (1.8) | 8 (2.9) | 40 (5.9) | 0.041 | 4.6 | 4.6 | 4.6 |
| Ustekinumab | 9 (2.3) | 14 (5.1) | 11 (1.6) | 0.035 | 2.4 | 2.5 | 2.4 |
| Guselkumab | 6 (1.5) | 0 (0.0) | 2 (0.3) | 0.015 | 0.7 | 0 | 0.5 |
| Secukinumab | 61 (15.6) | 69 (25.4) | 97 (14.2) | 0.111 | 17.2 | 17.3 | 17.2 |
| Ixekizumab | 41 (10.5) | 46 (16.9) | 51 (7.5) | 0.094 | 10.5 | 10.5 | 10.5 |
| Meteorological metrics during treatment^d^ |  |  |  |  |  |  |  |
| Temperature, °C | 20.6 (6.7) | 19.2 (9.3) | 16.5 (7.5) | 0.517 | 18.3 (7.0) | 18.3 (9.7) | 18.3 (7.7) |
| UV index | 9.3 (1.8) | 8.2 (2.8) | 6.1 (2.5) | 1.342 | 7.7 (2.1) | 7.7 (3.0) | 7.7 (2.7) |
| Humidity, % | 73.9 (5.0) | 76.4 (6.1) | 75.6 (4.7) | 0.478 | 75.3 (5.2) | 75.3 (5.9) | 75.3 (4.7) |

BMI, body mass index; NAFLD, nonalcoholic fatty liver disease; BMI, body mass index; PASI, Psoriasis Area and Severity Index; PGA, Physician’s Global Assessment; DLQI, Dermatology Quality of Life Index; UV, ultraviolet. Data are provided as mean (standard deviation) unless otherwise stated. ^a^After covariate balancing propensity score (CBPS) weighting, a single individual no longer represents a single data entity and thus raw counts are not reported after weighting. ^b^CBPS weighting provided precise balance of mean values of covariates (maximum absolute standardized difference < 0.001). ^c^The maximum absolute standardized difference observed among all pairwise comparisons. ^d^Mean monthly temperature, UV index, and humidity during the patient’s treatment period.
