# Supplementary material for: Impact of temperature trend-defined seasonality on psoriasis treatment outcomes: a multicenter longitudinal study
Source: Front Immunol. 2025 Sep 17;16:1641225. doi: 10.3389/fimmu.2025.1641225 (PMC12484154; doi:10.3389/fimmu.2025.1641225)
Supplement: Supplementary file 3 [file Table2.docx]

**Table S2** Number and frequency of missing records

|  | **3-month analysis** | | | **2-month analysis** | | |
| --- | --- | --- | --- | --- | --- | --- |
|  | **Warming** | **Transition** | **Cooling** | **Warming** | **Transition** | **Cooling** |
| Total | 313 | 548 | 550 | 391 | 272 | 681 |
| Age | 2 (0.6) | 3 (0.5) | 1 (0.2) | 1 (0.3) | 1 (0.4) | 2 (0.3) |
| Sex | 0 | 0 | 0 | 0 | 0 | 0 |
| BMI | 6 (1.9) | 6 (1.1) | 6 (1.1) | 4 (1.0) | 8 (2.9) | 8 (1.2) |
| College education | 9 (2.9) | 15 (2.7) | 18 (3.3) | 11 (2.8) | 12 (4.4) | 21 (3.1) |
| Smoker, ex and current | 0 | 0 | 0 | 0 | 0 | 0 |
| Disease duration | 2 (0.6) | 4 (0.7) | 3 (0.5) | 3 (0.8) | 3 (1.1) | 4 (0.6) |
| Family history | 0 | 0 | 0 | 0 | 0 | 0 |
| Psoriatic arthritis | 0 | 0 | 0 | 0 | 0 | 0 |
| Comorbidities |  |  |  |  |  |  |
| Cardiovascular disease | 0 (0.0) | 1 (0.2) | 1 (0.2) | 0 | 0 | 3 (0.4) |
| Diabetes | 0 (0.0) | 1 (0.2) | 1 (0.2) | 0 | 0 | 3 (0.4) |
| Hypertension | 0 (0.0) | 1 (0.2) | 1 (0.2) | 0 | 0 | 3 (0.4) |
| NAFLD | 0 (0.0) | 2 (0.4) | 1 (0.2) | 0 | 0 | 4 (0.6) |
| Hyperlipidemia | 0 (0.0) | 2 (0.4) | 1 (0.2) | 0 | 0 | 4 (0.6) |
| Hyperuricemia | 0 (0.0) | 2 (0.4) | 1 (0.2) | 0 | 0 | 4 (0.6) |
| Prior treatment |  |  |  |  |  |  |
| Biologics | 0 (0.0) | 4 (0.7) | 1 (0.2) | 2 (0.5) | 1 (0.4) | 2 (0.3) |
| Systemic nonbiologics | 0 (0.0) | 3 (0.5) | 1 (0.2) | 1 (0.3) | 1 (0.4) | 3 (0.4) |
| Phototherapy | 0 (0.0) | 2 (0.4) | 1 (0.2) | 1 (0.3) | 1 (0.4) | 0 |
| Exacerbation season |  |  |  |  |  |  |
| Spring | 4 (1.3) | 3 (0.5) | 1 (0.2) | 1 (0.3) | 1 (0.4) | 1 (0.1) |
| Summer | 4 (1.3) | 3 (0.5) | 1 (0.2) | 1 (0.3) | 1 (0.4) | 1 (0.1) |
| Autumn | 4 (1.3) | 3 (0.5) | 1 (0.2) | 1 (0.3) | 1 (0.4) | 1 (0.1) |
| Winter | 4 (1.3) | 3 (0.5) | 1 (0.2) | 1 (0.3) | 1 (0.4) | 1 (0.1) |
| Baseline PASI | 0 | 0 | 0 | 0 | 0 | 0 |
| Baseline PGA | 0 | 0 | 0 | 0 | 0 | 0 |
| Baseline DLQI | 13 (4.2) | 39 (7.1) | 34 (6.2) | 17 (4.3) | 27 (9.9) | 41 (6.0) |
| Treatment | 0 | 0 | 0 | 0 | 0 | 0 |
| Mean temperature | 0 | 0 | 0 | 0 | 0 | 0 |
| Mean UV index | 0 | 0 | 0 | 0 | 0 | 0 |
| Mean humidity | 0 | 0 | 0 | 0 | 0 | 0 |
| Outcome |  |  |  |  |  |  |
| PASI 75 | 0 | 0 | 0 | 0 | 0 | 0 |
| PASI 90 | 0 | 0 | 0 | 0 | 0 | 0 |
| PGA 0/1 | 1 (0.3) | 3 (0.5) | 1 (0.2) | 2 (0.5) | 2 (0.7) | 4 (0.6) |
| DLQI MID | 41 (13.1) | 89 (16.2) | 66 (12.0) | 46 (11.8) | 46 (16.9) | 85 (12.5) |

BMI, body mass index; NAFLD, non-alcoholic fatty liver disease; BMI, body mass index; PASI, Psoriasis Area and Severity Index; PGA, Physician’s Global Assessment; DLQI, Dermatology Quality of Life Index; UV, ultraviolet; MID, minimal important difference.
