# Supplementary material for: Impact of temperature trend-defined seasonality on psoriasis treatment outcomes: a multicenter longitudinal study
Source: Front Immunol. 2025 Sep 17;16:1641225. doi: 10.3389/fimmu.2025.1641225 (PMC12484154; doi:10.3389/fimmu.2025.1641225)
Supplement: Supplementary file 4 [file Table3.docx]

**Table S3** Response rates and odds ratios (ORs) for PASI 75, PASI 90, PGA 0/1, and DLQI MID at 2 months post-treatment

|  | **Unadjusted analysis** | | | **Adjusted analysis^a^** | | | |
| --- | --- | --- | --- | --- | --- | --- | --- |
|  | **Responders, n/N (%)^b^** | **OR (95% CI)^c^** | ***P*-value** | **Responders, % (95% CI)^c^** | **OR (95% CI)^c^** | ***P*-value** | ***P* for trend** |
| PASI 75 |  |  |  |  |  |  |  |
| Warming | 173/391 (44.2) | Ref |  | 43.5 (37.0, 50.1) | Ref |  |  |
| Transition | 142/272 (52.2) | 1.38 (1.01, 1.88) | **.044** | 41.3 (33.8, 48.8) | 0.92 (0.79, 1.07) | .298 |  |
| Cooling | 221/681 (32.5) | 0.61 (0.47, 0.78) | **<.001** | 35.6 (31.4, 39.8) | 0.73 (0.62, 0.85) | **<.001** | **<.001** |
| PASI 90 |  |  |  |  |  |  |  |
| Warming | 97/391 (24.8) | Ref |  | 23.3 (17.7, 28.9) | Ref |  |  |
| Transition | 75/272 (27.6) | 1.15 (0.81, 1.64) | .425 | 19.7 (13.6, 25.8) | 0.81 (0.67, 0.97) | **.022** |  |
| Cooling | 111/681 (16.3) | 0.59 (0.43, 0.80) | **<.001** | 19.8 (16.3, 23.4) | 0.81 (0.67, 0.98) | **.028** | **.029** |
| PGA 0/1 |  |  |  |  |  |  |  |
| Warming | 221/389 (56.8) | Ref |  | 58.9 (52.4, 65.4) | Ref |  |  |
| Transition | 152/270 (56.3) | 0.98 (0.71, 1.34) | .883 | 52.1 (44.5, 59.8) | 0.76 (0.65, 0.89) | **<.001** |  |
| Cooling | 339/677 (50.1) | 0.77 (0.60, 0.99) | **.038** | 52.6 (48.2, 57.0) | 0.79 (0.68, 0.93) | **.004** | **.005** |
| DLQI MID |  |  |  |  |  |  |  |
| Warming | 166/345 (48.1) | Ref |  | 50.7 (44.1, 57.3) | Ref |  |  |
| Transition | 140/226 (61.9) | 1.73 (1.24, 2.41) | **.001** | 53.0 (45.3, 60.6) | 1.20 (1.00, 1.44) | .057 |  |
| Cooling | 252/596 (42.3) | 0.77 (0.59, 1.00) | .052 | 48.1 (43.7, 52.6) | 0.87 (0.72, 1.06) | .159 | .134 |

PASI, Psoriasis Area and Severity Index; PGA, Physician’s Global Assessment; DLQI MID, Dermatology Quality of Life Index minimal important difference; CI, confidence interval. ^a^Balancing the exposure groups using covariate balancing propensity score (CBPS) weighting, adjusted for confounders including age, sex, BMI, disease duration, prior use of biologic and non-biologic systemic therapies, phototherapy, smoking status, family history of psoriasis, psoriatic arthritis, comorbidities (cardiovascular disease, diabetes, NAFLD, hypertension, hyperlipidemia, and hyperuricemia), baseline PASI, PGA, and DLQI scores, as well as mean temperature and humidity during treatment. ^b^As-observed results. ^c^Multiple imputation results. Significant *P*-values are highlighted in bold (*P* < 0.05).
