# Supplementary material for: Impact of temperature trend-defined seasonality on psoriasis treatment outcomes: a multicenter longitudinal study
Source: Front Immunol. 2025 Sep 17;16:1641225. doi: 10.3389/fimmu.2025.1641225 (PMC12484154; doi:10.3389/fimmu.2025.1641225)
Supplement: Supplementary file 6 [file Table5.docx]

**Table S5** Effectiveness in the sensitivity analyses at 2 months post-treatment

|  | **Sensitivity analysis 1^a^** | | **Sensitivity analysis 2^b^** | | **Sensitivity analysis 3^c^** | | **Sensitivity analysis 4^d^** | | **Sensitivity analysis 5^e^** | |
| --- | --- | --- | --- | --- | --- | --- | --- | --- | --- | --- |
|  | **Adjusted OR (95% CI)** | ***P*-value** | **Adjusted OR (95% CI)** | ***P*-value** | **Adjusted OR (95% CI)** | ***P*-value** | **Adjusted OR (95% CI)** | ***P*-value** | **Adjusted OR (95% CI)** | ***P*-value** |
| PASI 75 |  |  |  |  |  |  |  |  |  |  |
| Warming | Ref |  | Ref |  | Ref |  | Ref |  | Ref |  |
| Transition | 0.94 (0.78, 1.12) | .461 | 0.93 (0.79, 1.10) | .397 | 0.90 (0.76, 1.08) | .267 | 1.09 (0.91, 1.31) | .332 | 0.97 (0.82, 1.14) | .715 |
| Cooling | 0.76 (0.63, 0.92) | **.005** | 0.69 (0.59, 0.81) | **<.001** | 0.79 (0.66, 0.95) | **.013** | 0.78 (0.66, 0.92) | **.004** | 0.71 (0.60, 0.84) | **<.001** |
| PASI 90 |  |  |  |  |  |  |  |  |  |  |
| Warming | Ref |  | Ref |  | Ref |  | Ref |  | Ref |  |
| Transition | 0.78 (0.63, 0.96) | **.022** | 0.82 (0.68, 0.99) | **.039** | 0.88 (0.72, 1.08) | .215 | 1.55 (1.27, 1.88) | **<.001** | 0.80 (0.66, 0.98) | **.027** |
| Cooling | 0.80 (0.66, 0.97) | **.024** | 0.75 (0.62, 0.91) | **.004** | 0.84 (0.68, 1.03) | .099 | 0.93 (0.76, 1.12) | .436 | 0.72 (0.59, 0.88) | **.001** |
| PGA 0/1 |  |  |  |  |  |  |  |  |  |  |
| Warming | Ref |  | Ref |  | Ref |  | Ref |  | Ref |  |
| Transition | 0.73 (0.61, 0.88) | **<.001** | 0.79 (0.68, 0.94) | **.006** | 0.87 (0.68, 1.11) | .258 | 0.93 (0.77, 1.13) | .479 | 0.81 (0.69, 0.96) | **.014** |
| Cooling | 0.85 (0.71, 1.03) | .094 | 0.81 (0.69, 0.95) | **.009** | 0.84 (0.68, 1.03) | .094 | 0.87 (0.74, 1.03) | .102 | 0.80 (0.68, 0.95) | **.010** |
| DLQI MID |  |  |  |  |  |  |  |  |  |  |
| Warming | Ref |  | Ref |  | Ref |  | Ref |  | Ref |  |
| Transition | 1.30 (1.06, 1.61) | **.015** | 1.19 (0.98, 1.44) | .077 | 1.21 (0.97, 1.50) | .100 | 0.66 (0.49, 0.91) | **.014** | 1.20 (0.98, 1.46) | .075 |
| Cooling | 0.97 (0.64, 1.45) | .869 | 0.84 (0.69, 1.02) | .084 | 0.87 (0.71, 1.06) | .174 | 0.90 (0.73, 1.12) | .341 | 0.84 (0.69, 1.04) | .111 |

OR, odds ratio; CI, confidence interval; PASI, Psoriasis Area and Severity Index; PGA, Physician’s Global Assessment; DLQI MID, Dermatology Quality of Life Index minimal important difference. ^a^Sensitivity analysis 1: Replacing mean temperature with mean UV index during treatment in the CBPS model. ^b^Sensitivity analysis 2: Truncating CBPS weights at the 5^th^ and 95^th^. ^c^Sensitivity analysis 3: Excluding patients undergoing phototherapy. ^d^Sensitivity analysis 4: Excluding patients exposed to mean temperatures ≤ 10^th^ percentile. ^e^Sensitivity analysis 5: Excluding patients with baseline PASI scores in the ≥ 90^th^ percentile.
