# Supplementary material for: Impact of temperature trend-defined seasonality on psoriasis treatment outcomes: a multicenter longitudinal study
Source: Front Immunol. 2025 Sep 17;16:1641225. doi: 10.3389/fimmu.2025.1641225 (PMC12484154; doi:10.3389/fimmu.2025.1641225)
Supplement: Supplementary file 7 [file Table6.docx]

**Table S6** Interaction analyses of temperature trends (the cooling group vs. the warming group) with predictors for PASI 75 at 2 months

|  | **Interaction of ORs^a^** | | **Interaction of RDs^b^** | |
| --- | --- | --- | --- | --- |
| **Predictor^c^** | **Interaction (95% CI)** | ***P*-value** | **Interaction (95% CI)** | ***P*-value** |
| Age (49.0 years) | 0.86 (0.47, 1.59) | .633 | -0.032 (-0.136, 0.073) | .553 |
| Male sex | 1.20 (0.60, 2.40) | .599 | 0.039 (-0.081, 0.160) | .523 |
| Disease duration (12.0 years) | 0.87 (0.47, 1.60) | .658 | -0.030 (-0.135, 0.074) | .568 |
| BMI (24.5) | 0.29 (0.16, 0.54) | **<.001** | -0.206 (-0.310, -0.101) | **<.001** |
| Baseline PASI (12.0) | 0.88 (0.48, 1.61) | .670 | -0.032 (-0.137, 0.073) | .553 |
| Baseline DLQI (9.0) | 0.87 (0.47, 1.60) | .656 | -0.030 (-0.134, 0.075) | .579 |
| Baseline PGA 3, 4 vs 1, 2 | 0.59 (0.32, 1.11) | .101 | -0.095 (-0.201, 0.011) | .080 |
| Mean temperature during treatment (19.7 °C) | 1.24 (0.66, 2.33) | .507 | 0.038 (-0.071, 0.147) | .491 |
| Mean humidity during treatment (73.8%) | 0.67 (0.36, 1.26) | .213 | -0.065 (-0.172, 0.042) | .233 |
| Mean UV index during treatment (7.0) | 2.00 (0.78, 5.14) | .151 | 0.127 (-0.040, 0.294) | .136 |
| Treatment |  |  |  |  |
| Methotrexate | 0.88 (0.14, 5.54) | .895 | -0.045 (-0.218, 0.128) | .611 |
| Phototherapy | 0.48 (0.08­, 3.00) | .432 | -0.161 (-0.330, 0.009) | .064 |
| Adalimumab | 1.31 (0.10, 16.98) | .834 | 0.040 (-0.324, 0.404) | .830 |
| Ustekinumab | 6.76 (0.46, 100.32) | .165 | 0.401 (-0.009, 0.794) | .055 |
| Guselkumab | 1.28 (0.03, 53.89) | .898 | 0.024 (-0.664, 0.712) | .945 |
| Secukinumab | 0.39 (0.06, 2.77) | .349 | -0.173 (-0.366, 0.020) | .080 |
| Ixekizumab | 0.47 (0.06, 3.70) | .473 | -0.134 (-0.355, 0.087) | .235 |
| Smoker | 1.37 (0.73, 2.59) | .332 | 0.054 (-0.070­, 0.179) | .392 |
| Family history | 1.04 (0.49, 2.18) | .925 | 0.004 (-0.141, 0.149) | .956 |
| College education | 0.73 (0.38, 1.38) | .326 | -0.052 (-0.177, 0.072) | .410 |
| Prior biologics | 1.33 (0.46, 3.85) | .597 | 0.050 (-0.153­, 0.253) | .629 |
| Prior nonbiologics | 1.14 (0.58, 2.24) | .701 | 0.027 (-0.104, 0.158) | .687 |
| Prior phototherapy | 1.37 (0.72, 2.62) | .336 | 0.061 (-0.065, 0.187) | .344 |
| Exacerbation season |  |  |  |  |
| Spring | 0.92 (0.35, 2.43) | .868 | -0.013 (-0.181, 0.155) | .879 |
| Summer | 1.16 (0.33, 4.06) | .819 | 0.011 (-0.214, 0.236) | .923 |
| Autumn | 0.86 (0.35, 2.11) | .740 | -0.026 (-0.184, 0.131) | .742 |
| Winter | 1.15 (0.62, 2.13) | .658 | 0.024 (-0.082­, 0.130) | .653 |
| Psoriatic arthritis | 3.47 (0.64, 8.52) | .501 | 0.191 (-0.082, 0.416) | .254 |
| Comorbidities |  |  |  |  |
| Cardiovascular disease | 0.69 (0.18, 2.65) | .585 | -0.069 (-0.305, 0.166) | .564 |
| Diabetes | 1.62 (0.65, 4.01) | .300 | 0.081 (-0.078, 0.239) | .318 |
| Hypertension | 0.97 (0.49, 1.92) | .923 | -0.015 (-0.135, 0.105) | .810 |
| NAFLD | 0.58 (0.21, 2.84) | .702 | -0.098 (-0.271, 0.075) | .265 |
| Hyperlipidemia | 1.03 (0.36, 2.94) | .654 | 0.009 (-0.176, 0.193) | .926 |
| Hyperuricemia | 0.68 (0.20–2.27) | .533 | -0.074 (-0.287, 0.139) | .495 |

PASI, Psoriasis Area and Severity Index; OR, odds ratio; RD, risk difference; CI, confidence interval; BMI, body mass index; PGA, Physician’s Global Assessment; DLQI MID, Dermatology Quality of Life Index minimal important difference; NAFLD, non-alcoholic fatty liver disease. ^a^Defined as the ratio of ORs which compares the ORs in patients in the cooling group vs. the warming group across the specified subgroups. ^b^Defined as the difference in RDs, which is the absolute difference in RDs in patients in the cooling group vs. the warming group across the specified subgroups. ^c^Continuous variables are dichotomised at the median. Significant *P*-values are highlighted in bold (*P* < 0.05).
